# Maximum Entropy Reconstructions of Dynamic Signaling Networks from Quantitative Proteomics Data

**DOI:** 10.1371/journal.pone.0006522

**Published:** 2009-08-26

**Authors:** Jason W. Locasale, Alejandro Wolf-Yadlin

**Affiliations:** 1 Division of Signal Transduction, Department of Systems Biology, Harvard Medical School, Beth Israel Deaconess Medical Center, Boston, Massachusetts, United States of America; 2 Department of Chemistry and Chemical Biology, Harvard University, Boston, Massachusetts, United States of America; National Cancer Institute, United States of America and Tel Aviv University, Israel

## Abstract

Advances in mass spectrometry among other technologies have allowed for quantitative, reproducible, proteome-wide measurements of levels of phosphorylation as signals propagate through complex networks in response to external stimuli under different conditions. However, computational approaches to infer elements of the signaling network strictly from the quantitative aspects of proteomics data are not well established. We considered a method using the principle of maximum entropy to infer a network of interacting phosphotyrosine sites from pairwise correlations in a mass spectrometry data set and derive a phosphorylation-dependent interaction network solely from quantitative proteomics data. We first investigated the applicability of this approach by using a simulation of a model biochemical signaling network whose dynamics are governed by a large set of coupled differential equations. We found that in a simulated signaling system, the method detects interactions with significant accuracy. We then analyzed a growth factor mediated signaling network in a human mammary epithelial cell line that we inferred from mass spectrometry data and observe a biologically interpretable, small-world structure of signaling nodes, as well as a catalog of predictions regarding the interactions among previously uncharacterized phosphotyrosine sites. For example, the calculation places a recently identified tumor suppressor pathway through ARHGEF7 and Scribble, in the context of growth factor signaling. Our findings suggest that maximum entropy derived network models are an important tool for interpreting quantitative proteomics data.

## Introduction

The principles that underlie how mammalian cells detect, integrate, and utilize external signals to achieve an appropriate phenotypic response are a subject currently of intense study[1]. Signals mediated by receptor tyrosine kinases (RTKs), such as stresses, growth factors, antigens, among many others, lead to the phosphorylation of hundreds of tyrosine residues leading to conformational changes that allosterically regulates interactions with specific binding partners[2], [3]. Such regulation cooperates to form complex biochemical signaling networks[4]. Recent technological advances in the application of mass spectrometry and array-based methods to phosphoproteomics have allowed for the quantitative measurement, under different conditions, of the relative activities of hundreds of tyrosine residues as they undergo reversible phosphorylation in response to a stimulus[5], [6], [7], [8], [9].

The enormity of quantitative data acquired in these experiments raises the question of what modeling approaches might be used to lend predictive and mechanistic insight into the signaling networks that govern the behavior of these phosphorylation sites[10]. Clustering and other measures of correlation have successfully grouped large data sets including data derived from mass spec-measured signaling dynamics into similar patterns[5]. Partial least squares regression modeling (PLSR) has also been employed to identify what aspects of these data sets are most correlated with different phenotypic responses[7], [11]. These statistical techniques have shown to be very useful in their predictive capabilities and have yielded new biological insights[12].

Despite these many advances, computational approaches for inferring the actual interaction networks from quantitative, condition dependent activities in the proteomics data have not been fully investigated. Methods for network inference such as Bayesian approaches[13] or mutual information[14] require an knowledge of a full distribution of activities across an ensemble of measurements at each node in the network. As such, these methods are not applicable when the number of samples is small (e.g. ∼10 sample conditions for typical Mass spectrometry or protein-array experiments).

Also, quantification at each phosphorylation site is subject to many sources of error that are hard to account for, and it is not clear a priori the level of quantitative detail that such experiments provide. Therefore, the question that we aim to address is the following: assuming no prior information as to the network of causal activities among the measured set of phosphorylation sites, to what extent can phosphoproteomics, through a series of quantitative measurements that monitor the activity of phosphorylation sites, be used to infer a signaling network (in which the activities of each site are embedded in an interaction network)?

To address this question, we consider the least biased model that incorporates only the statistics of the correlation in phosphorylation levels at different phosphorylation sites under different conditions (as measured by mass spectrometry). We use these correlations to constrain a model of pairwise interactions between phosphorylation sites that constitute a full signaling network. Such a model is obtained from information theoretic considerations by applying the principle of maximum entropy(methods)[15].

Models of pairwise network connectivity obtained from entropy maximization have proven insightful in different and seemingly disparate contexts[16], [17]. Bialek and coworkers applied the principle of entropy maximization to construct an interaction network of neurons that respond to visual stimuli[17]. In another example, Fedoroff and coworkers use the principle to derive a genetic interaction network from microarray data in yeast[16].

However, one major difference in cell signaling systems from these previously studied systems is that phosphorylation patterns display transient, non-stationary behavior that makes the notion of a statistical ensemble unclear. Furthermore, the structure of phosphorylation networks are likely to be fundamentally different from those involving gene expression. Therefore, we first investigated the applicability of this approach to cell signaling. We applied the entropy maximization principle to a simulated biochemical signaling network with non-stationary dynamics and known network connectivity. We find that in this model system whose quantitative signal outputs are governed by a large set of coupled nonlinear differential equations, the method detects known interactions with significant accuracy. We then analyzed a proteome-wide mass spectrometry data set[8] of a growth factor signaling network in a human mammary epithelial cell line and observed a biologically interpretable, small-world structure of interacting signaling nodes. We also derive a set of predictions regarding the interactions among previously uncharacterized signaling nodes. Our approach suggests that signaling networks inferred solely from quantitative proteomics data generate many novel biological hypotheses and are a useful tool for interpreting large quantitative proteomics data sets.

Furthermore, upon inspection of our calculated signaling network, we observe new mechanistic features of the growth factor mediated signaling network. For example, we identify the effector ARHGEF7 of a recently characterized tumor suppressor protein, Scribble[18], as a key node within the network and place its activity in the context of other known regulators of growth factor signaling. Our computation also identifies the LDL receptor, previously not known to function in growth factor signaling, as having a possible role in coordinating the activity of the epidermal growth factor receptor (EGFR). Also the study predicts the function of a novel pleckstrin-homology (PH) domain containing protein, PI3BP, and its possible role in lipid kinase secondary messenger signaling.

## Results

### Maximum entropy principle applied to phosphorylation site interaction networks

From the correlations in the time courses, we seek to indentify which phosphorylation sites affect the activities of other phosphorylation sites and whether this activity positively or negatively affects its targeted phosphorylation site. An interaction in such a network is thus interpreted as a pair of phosphorylation sites whose activities are most closely related. The relation can result from a series of indirect interactions such as one phosphorylation site recruiting a kinase that phosphorylates another site in the network. Other scenarios that lead to an interaction are also possible such as a phosphorylation event affecting the recruitment of a protein to a membrane or a scaffold that results in its own phosphorylation or desphosphorylation by a separate effector. For example, phosphoinositide 3-kinase (PI3K) catalyzes the conversion of the lipid product PIP2 to PIP3 and subsequent PIP3 binding at cell membranes by pleckstrin-homology (PH) domain-containing proteins leads to phosphorylation or desphosphorylation of the corresponding PH domain containing protein such as AKT which then has over 100 downstream targets[19]. In this situation, a phosphorylation site on PI3K could interact with a downstream substrate of AKT. The inferred network could then be used to interpret the local, dynamic biological function of different phosphorylation sites as they undergo reversible covalent modifications.

Our aim is to arrive at such a network using only the quantitative activities in the proteomics data. From the computation, we aim to extract as much information as possible from a proteomics experiment. We do not expect to recover a full set of phosphorylation-dependent interactions; rather, we investigate the extent to which the least biased partial network derived strictly from quantitative proteomics data provides useful information.

Assuming no prior data, an unbiased network model consists of independent signaling nodes whose activities are uncorrelated. However, the quantitative measurements in mass spectrometry data contain information about the structure of the network and correlated activity profiles between nodes. Pairwise correlations averaged over the quantitative values of phosphorylation site activity would then give rise to a model of interactions in which the activity at each site is dependent upon a network of phosphorylation sites with interactions between each node.

Given the activities of each signaling node and their pairwise correlations, the principle of maximum entropy gives the probability of having a particular network configuration (i.e. set of relative amounts of phosphorylation at each signaling node) 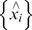, 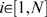 (methods):
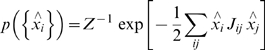
(1)


Where 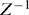
is a normalization factor that is not considered for the purpose of this study. Important to note is that this distribution can also be obtained from other arguments[16]. We emphasize however that this is the maximum entropy distribution to underscore the point that the model, and interactions encoded within it, is mathematically the least biased attempt at the inference of a phosphorylation site interaction network. Therefore, to the extent that useful information can be obtained from this model is indicative of the utility of such mass spectrometry data to encode an interaction network.

In this model, 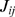 are the elements of the resulting interaction matrix that defines the pairwise network connectivity between the i^th^ and j^th^ phosphorylated tyrosine site and is the inverse of the element in the corresponding correlation matrix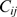,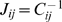. Each value is mean centered and normalized to unit variance, 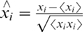, where the brackets denote an average over the set of measurements and *X_i_* is the bare value of phosphorylation at the i^th^ signaling node. This is done in part because the relationships among the relative magnitudes of phosphorylation activities (at different sites) are poorly resolved in phosphoproteomics data. As a result, phosphorylation sites having a small variance across the different conditions (on the order of the error bars in the measurements) were not used in the calculation.

From the original data, it is noted that there are many more phosphorylation sites than number of time points sampled 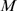 (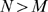), and as a result, 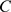 is singular (i.e. the rank of *C* is not complete). Therefore, 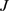 is obtained by inverting *C* in the space of non-zero eigenvalues. First, 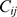 is expanded in terms of its eigenvalues and coefficients of its eigenvectors: 
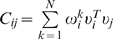
, where the superscript 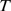 denotes the transpose of the eigenvector 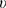. The matrix element 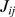 is obtained by inverting *C* in the space of eigenvectors containing non-zero eigenvalues by considering only the non-zero eigenvalues of 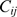:
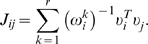
(2)



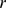
 is the rank of the covariance matrix.

It is important to note that the interaction at sites i and j is not necessarily revealed by the correlation since other long-range global influences may dominate the correlations. From the expansion, it is seen that the correlations are dominated by the largest eigenvalues whereas the interactions that constitute the network connectivity are dominated by the smallest eigenvalues. Therefore, a key feature of the inferred maximum entropy network is that the matrix elements 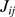 constitute the residual, pairwise interactions between phosphorylation sites i and j that remain once global effects that are dominated by the correlations (i.e. the largest eigenvalues) are effectively removed[16]. Other information theoretic methods that infer biological network connectivity from data sets with larger sample sizes[14], also involve a considerable effort to remove effects that are dominated by global correlations such as the use of the data-processing inequality[15]. In the Maximum Entropy approach, long-range correlations that influence the activity are naturally removed by deemphasizing the contributions of large eigenvalues in the correlation matrix.

The interaction matrix 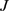 (Fig. S1) encodes a total of 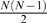 pairwise interactions among the phosphotyrosine sites. Fig. S2 contains a scatter plot of the elements of 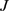 plotted against the elements of *C*. For 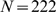 sites, 24,531 interactions are possible. However, the histogram of 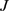 values computed from the data is sharply peeked at zero (Fig. S1a) indicating that most entries in the matrix contribute little to the network. From eq. 2 the diagonal entries of 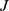 indicate the “self” interactions of the network and are plotted in Fig. S3. Large values indicate the phosphorylation sites that contribute most to the structure of the network and can be interpreted as network “hubs”.

Therefore, a parameter that defines a threshold value of interaction strength is introduced and different networks are obtained for different threshold values. For a given value of threshold interaction strength, *T*, an interaction between phosphorylation sites at positions i and j is counted if the magnitude of 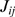 exceeds the threshold; 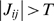. A connectivity matrix
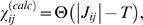
(3)where 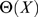 is a step function that equals 1 if 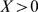 and 0 otherwise, is used to define the network connectivity. Since the choice of threshold *T* is arbitrary, each subsequent calculation must be carefully considered with respect to its dependence on the value of *T*. Fig. S4 illustrates how the connectivity of the network changes for different choices of *T*.

### Entropy maximization principle of network connectivity in a simulated signal transduction network

It is important to note that measured time courses (whose data points in this case constitute the different samples) from the mass spectrometry data (and signal transduction in general) are not stationary; a further complication arises from the fact that the measurements under different conditions (i.e. across time) are not necessarily uncorrelated. As a result, the physical meaning of the ensemble derived from maximizing the entropy functional is not clear. Therefore, it is not understood, a priori, to what extent does a model constrained to pairwise correlations in the mass spec data captures known interactions.

To begin to understand the utility of the method, we investigated the applicability approach by simulating the dynamics of a model signaling network with known network topology. A model of a signaling cascade was considered and an ordinary differential equation (ODE) model was used. We focused on this model because the model consists of a transient response within a complex signaling network with nonlinear feedback loops and many interconnected chemical reactions. Most importantly, the simulated time courses in this model display a similar pattern, characterized by an overall rise and subsequent decay of signaling intermediates over time, of activities to that observed in the mass spectrometry data. The published signaling model consists of G-coupled protein receptor signaling leading to myosin light chain phosphorylation[20] and was obtained from the Biomodels database[21]. The model consists of a set of 105 coupled ODEs and 110 half reactions.

**Figure 1:**
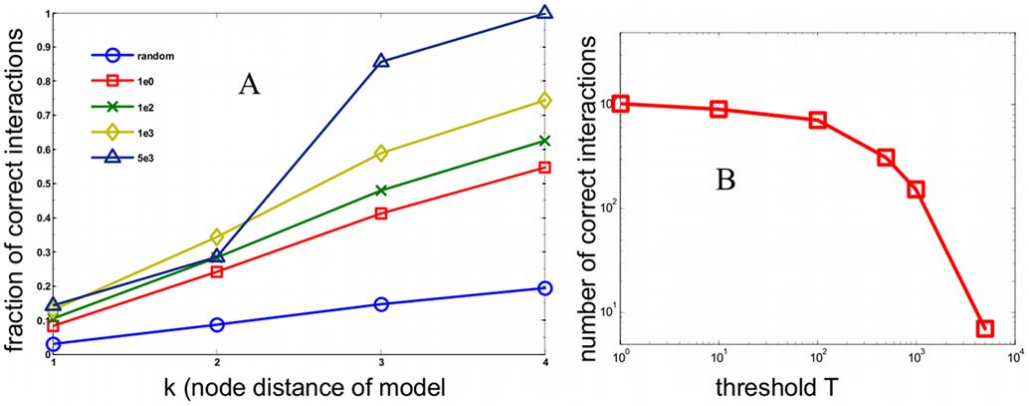
Application of the pairwise Maximum Entropy model to network structure to a simulated biochemical signaling model. Model consists of 105 chemical species coupled through 110 chemical reactions. Full details and parameters are available in the Biomodels database[21] and ref. [20]. a.) Accuracy measure: fraction of correct interactions as a function of node distance for different threshold values are considered. Different thresholds used are compared to a random network of 105 chemical species. Red line denotes the expected value for a network of random connections. b.) Specificity measure: total number of correctly detected interactions as a function of threshold.

We first investigated the accuracy of the inferred network. For each choice of threshold, the network outperforms the expected value obtained a network with uniformly chosen random bonds (blue, circles). Fig. 1a considers the fraction of correction interactions (defined in the supplementary information). As the threshold is increased (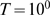 (red, squares), 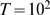 (green, crosses), 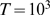 (yellow, diamonds)), and 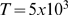 (violet, triangles)), the inferred network detects real interactions with high accuracy and significantly outperforms the random network by many factors at node distances (defined in methods) of 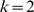, 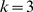, and 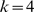. Note the computation does not perform well for 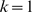 because of the convention chosen for our definition of node distance (methods). For high threshold values, the calculated network achieves perfect accuracy at larger node distances. To study the specificity of the calculated network, we plotted the total number of correct interactions detected as a function of *T* in Fig. 1b. From Fig. 1b, it is apparent that the maximum entropy network provides significant coverage of the full network derived from the stoichiometry matrix of the simulated model (methods). Of the 110 total half reactions, the calculated network detects this many interactions at roughly a threshold value of 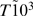 corresponding to an accuracy of ∼75% at a node distance of 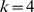.

### Application of a maximum entropy network model to a quantitative phosphoproteomic data set

**Figure 2:**
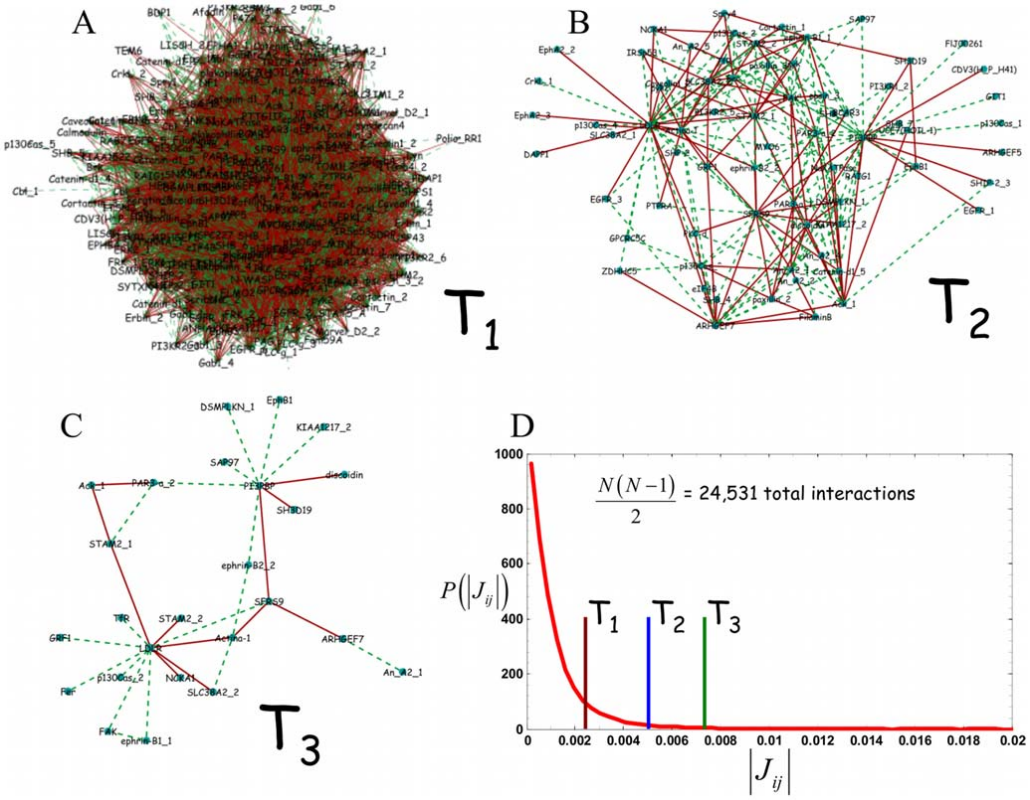
Threshold dependent phosphorylation interaction networks. Graphical depictions of inferred phosphotyrosine interaction networks at different thresholds. Green lines denote positive connections and red lines denote negative connections. Networks at three thresholds are shown: a.) 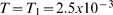, b.) 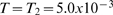, and c.) 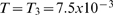 d.) Histogram of the magnitude of the 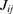 interactions and the relative location of the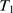, 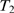, and 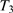 cutoffs.

Having applied the maximum entropy approach to network connectivity in a simulated cell signaling system, we then considered its application to a proteomic data set. Fig. 2 considers graphical depictions of the phosphorylation interaction network at different thresholds obtained from eq. 3. At a low threshold (Fig. 2a), 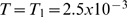, the network is not easily interpretable. At intermediate threshold (Fig. 2b), 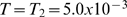, the network can be visualized as containing a core structure of nodes. At a high threshold (Fig. 2c), 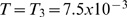, a small set of interconnected nodes is present. Fig. 2d shows the relative location of each threshold within the histogram of all 24,531 interactions. From the histogram, it is apparent that the distribution of the magnitude of 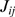 is sharply peaked at zero with the higher threshold choices containing only a small fraction of the total number of interactions. The nodes consist of a set phosphorylation sites appear to be connected is closely connected to each other site more than would be expected in a random network[22].

**Figure 3:**
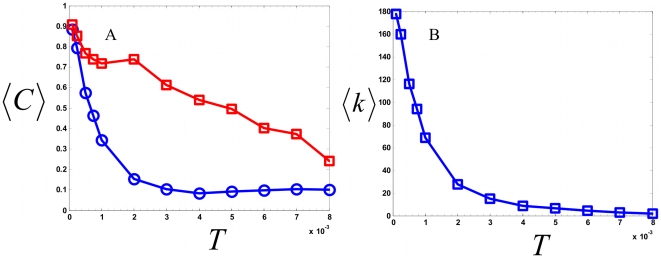
Clustering properties of the inferred signaling networks. Topological properties of inferred signaling networks as compared to those of a random network. a.) Average clustering coefficient 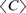 is plotted against different values of interaction threshold *T*. Maximum entropy network (red, squares) and a random network with the given number of nodes and edges of inferred network (blue, circles) are considered. b.) mean number of nearest nodes 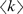 plotted against the interaction threshold *T*.

Since it is difficult to compare the calculated network in a quantitative sense with known signaling pathways, we considered quantitative measures of network structure and compared these measures to those expected from a random network. One way of characterizing the network is to compute an average clustering coefficient 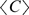 [22]. We expect the observation of deviations of this quantity away from that expected in a random network to be indicative of local structure within the network and also suggestive of the degree to which the network contains useful information in its correlated, clustered connections. This structure could be interpreted as the existence of local groups or modules of signaling nodes whose activities are coordinated. The clustering coefficient at node i, 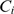, defined as 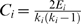, is the number of connections 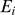 between nearest neighbors in a network with 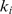 nearest neighbors divided by the number of possible connections. Fig. 3a shows a plot of the calculated value 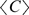 (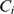 is averaged over each node) as a function of threshold *T* (red, squares). The curve is compared to what would be expected from a random network with as many nodes and edges (blue, circles) (methods). As seen in Fig. 3a, at small values of *T*, the computed value of 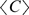 is nearly indistinguishable from that of a random network. This property likely defines the point at which *T* is sufficiently low that the network too noisy to be interpretable. For intermediate values of *T*, the computed networks have significantly higher values of 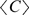 than would be expected of a random network. At this value, the model provides a highly correlated network structure. Finally, at large values of *T*, few nodes and edges are available to form a network and as a consequence, the calculated 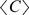 also deviates less from the expected value for a random network. As a reference to the size of the network, Fig. 3b plots the average number of nearest neighbors 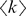 as a function of *T*. From the plot in Fig. 3b, it is apparent that for values of *T* that lead to a high value of 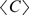, a sufficient number of interactions are detected to form a coherent network.

### Biological interpretation of the network

**Figure 4:**
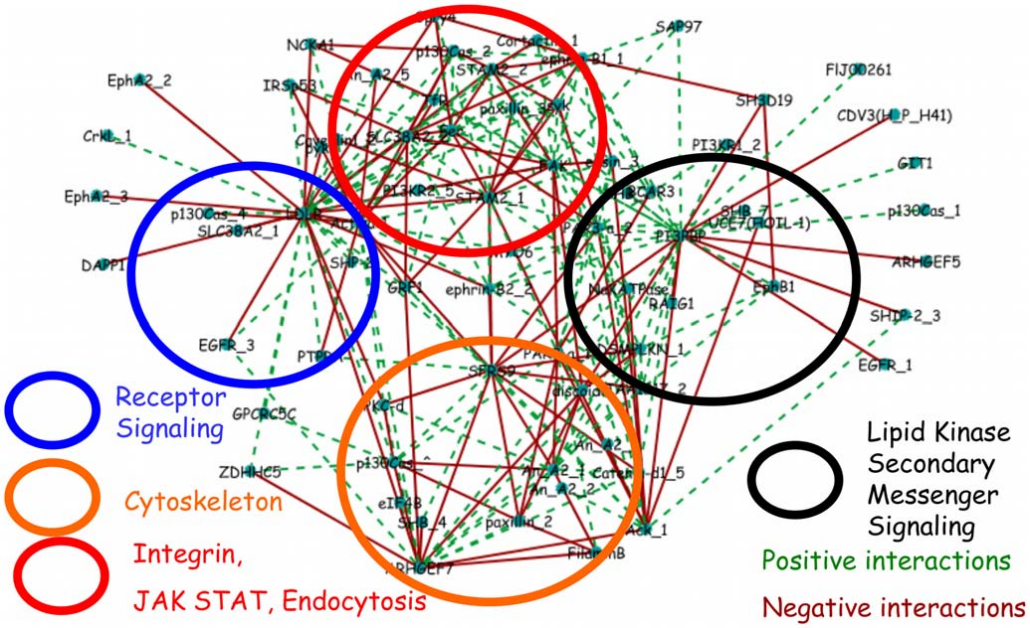
Inferred interaction network at intermediate threshold gives a modular, biologically interpretable signaling network. Graphical depiction of the signaling network at intermediate (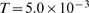) threshold. (Tyrosine phosphorylation sites are grouped, for visualization, into four functional categories: receptor signaling (blue circle), cytoskeleton (orange circle), lipid kinase secondary messenger signaling (black circle), and integrin, JAK/STAT signaling, and endocytosis (red circle). Green connections denote positive and red connections denote negative interactions. Full annotation for each abbreviated site name shown in the graph is given in the supplementary information

An inspection of the network at an intermediate threshold (Fig. 4) reveals connected phosphotyrosine sites of disparate functional significance. The core structure of the signaling network contains phosphorylation sites on proteins involved, in endocytosis, gene splicing, Mitogen-activated protein kinase (MAPK) signaling, PI3K signaling among others.

In Fig. 4, a network consisting of distinct structures involving elements of the growth factor signaling network is observed. This behavior is further quantified in Fig. S4 which considers the node distribution at different thresholds. We grouped the interconnected phosphorylation sites into four categories. One set (blue circle) consists largely of receptor and membrane proximal signaling and comprises the LDL receptor (an EGF binding domain-containing protein that plays a role in lipid transport in epithelial cells), an Epidermal growth factor receptor (EGFR) phosphorylation site, Epithelial Cell Receptor A2 (EPHA2), a receptor tyrosine kinase that also activates canonical downstream effector pathways), among others. Another group (black circle) consists of many phosphorylation sites known to be involved in lipid kinase secondary messenger signaling such as the PI3K pathway[19]. These phosphorylation sites include sites on PI3BP, the 5′ inositol phosphatase SHIP2, and PIK3R, the p85 regulatory subunit of PI3K. Another set (red circle) contains phosphorylation sites involved in processes immediately downstream of receptor activation such as endocytosis, integrin, and Jak/Stat signaling. These phosphorylation sites involve proteins having a number of functions in Endocytosis (e.g. STAM2), a phospholipase Annexin A2, and Caveolin. The other set of nodes (orange circle) contains many phosphorylation sites associated with cytoskeletal dynamics such as paxillin, filaminB, as well as SFRS9, a putative alternative splicing factor. The choice of these groupings was made to facilitate the biological interpretation of the network.

### Novel features of the growth factor signaling network

An inspection of the diagonal elements 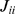 of the interaction matrix contains information about the ‘self’ interactions of the network (Fig. S3). These elements 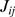 are a measure of the overall contribution of the i^th^ phosphorylation site to the structure of the network. The sites with the largest self interaction can be considered the network hubs. Table 1 contains a list of the phosphorylation sites with the 10 largest self interactions. These hubs determine the core structure of the signaling network.

**Table 1:** Highest scoring phosphotyrosine sites.

Name	Self Interaction (x10̂-2)	(Variance)^-1 (x10̂2)
PI3BP_Y492	2.51	7.06
LDLR	1.55	2.14
ARHGEF7	1.14	7.33
PAR3aY1127	1.11	2.02
PAR3aY1080	1.09	9.61
FAK	1.03	1.06
Actin-a1	1.03	5.88
ACK_Y857/Y858	1.2	0.06
SFRS9	1.01	4.98
STAM2_Y292	0.94	0.03

Functional aspects of the signaling network are apparent from inspection of the high scoring phosphorylation sites. In the case of receptor-mediated signaling, the LDL receptor is shown to coordinate its activity with EGFR which also interacts with sites on the Ephrin receptor. These receptors may be acting in concert to coordinate signals to other areas of the pathway. In another region of the network, lipid kinase secondary messenger signaling is regulated by a series of interactions involving the regulatory subunit of PI3-Kinase (PI3K), a little characterized 3′ phosphoinositol binding protein PI3PBP, and an inositol phosphatase (SHIP-2) that is known to be critical in regulating signaling through PI3K. Although the coordinated regulation of PI3K and SHIP-2 is well documented[19], PI3PBP and its interactions are unknown. Other regions of the network also contain novel functional predictions.

### Previously uncharacterized signaling nodes

Of the 222 detected tyrosine-phosphorylated peptides that comprise the signaling network in the data set that we used, 31 have been previously unassociated with the EGFR signaling network[8]. The interaction matrix also makes predictions about the connectivity of the uncharacterized nodes. Table 2. lists the 5 largest self interactions among the phosphorylation sites that have not been associated with the network.

**Table 2:** Highest scoring previously uncharacterized phosphotyrosine sites.

Name	Self Interaction (x10̂-2)	(Variance)^-1 (x10̂2)
ARHGEF7	1.14	7.33
SH3D19	0.7	1.26
ZDHHC5	0.55	2.87
An A2	0.49	2.73
GPCRC5C	0.45	2.77

The highest scoring uncharacterized phosphorylation site is associated with a protein ARHGEF7. ARHGEF7 is a mediator of the Tumor suppressor, Scribble[18], associated with the Rho GTPase Rac and its pathway[23]. The Rac pathway is involved in cytoskeletal rearrangement and cell motility among many other functions. The model predicts that ARHGEF7 coordinates receptor signaling with cytoskeletal proteins and also makes interactions with a splice factor SFRS9 which can regulate alternate splicing events. In light of this finding, it is also interesting speculate that SFRS9 may be regulating splicing events that coordinate cytoskeletal processes.

The next highest scoring uncharacterized phosphorylation site is associated with a poorly characterized SH3 domain containing protein SH3D19. SH3D19 is a likely adapter protein that contains five SH3 domains[24]. The third highest scoring phosphorylation site is associated with a zinc finger domain containing protein ZDHHC5. ZDHHC5 was identified in an shRNA screen for genes involved in the division of HeLa cells[25]. The next highest scoring phosphorylation site is associated with a protein known as Annexin A2. Annexin A2 is a phospholipase family member and has numerous functions including endocytosis and the generation of lipid secondary messengers[26]. Finally, the fifth highest scoring phosphorylation site is associated with GPCRC5C. GPCR5C is a tissue specific, G-protein coupled receptor[27]. Fig. S5. contains a graphical representation of the network connectivity of the three highest scoring phosphorylation sites, those associated with: ARHGEF7, SH3D19, and ZDHHC5.

## Discussion

We used the maximum entropy principle to infer a network of interactions between phosphorylation sites assuming only constraints obtained from pairwise correlations observed in quantitative mass spectrometry data. As a result, we were able to obtain, for the first time from phosphoproteomics mass spectrometry data, a biologically interpretable signaling network that predicts the interactions involved with previously uncharacterized signaling nodes. For a sufficiently high threshold value of interaction strength, a small-world network topology is observed in which the average clustering coefficient is much larger than would be expected for a random network[22]. This core structure of the derived network connectivity contains many known signaling intermediates with previously characterized interactions along with poorly uncharacterized tyrosine phosphorylation sites and their predicted interactions. It is also important to note that because the network depends on a threshold parameter, a decrease in the number of false positives will necessarily accompany an increase in the number of false negatives (true interactions that are not detected). Thus, the network that we compute at high threshold values, although likely accurate, is by no means complete. Nonetheless, such a model of network connectivity serves as a resource for the biological community in generating new hypotheses on the nature of signal transduction mediated by phosphorylation networks.

Unfortunately, mass spectrometry and other proteomics data are inherently noisy and it is difficult to account for sources of noise that affect the quantitation. Invariably, these errors are propagated into our calculation in ways that are uncontrolled. Also, the amount of information that can be obtained from these experiments is fundamentally limited by the small number of measurements at each node in the network and the maximum entropy approach merely intends to extract the optimal amount of information from each measurements. Despite these difficulties, a network with biological interpretability was inferred solely from correlations in the quantitative mass spectrometry data.

## Methods

One key assumption in developing the model is that the relative amounts of Tyrosine phosphorylation measured at each phosphorylation site for each time point constitute one sample from a statistical ensemble of possible phosphorylation states whose activities fluctuate on an interconnected network. Since time courses are measured up to a time of approximately one half hour, the differences in phosphorylation levels measured at each time point arise from changes in the amount of reversible post translational modifications. Changes in gene expression occur on longer time scales[28] so we do not expect the time courses to be affected by gene transcription upon which the upregulation of genes will affect the network topology. Therefore, it is reasonable to expect the same protein-protein interaction network to be present across a time scale of 30 minutes.

We characterize the state of the system with a state vector 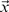 that contains the relative amount of pY phosphorylation at each measured site;
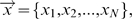
(S1)



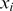
 is the amount of phosphorylation at the i^th^ phosphorylation site.

A probability of observing the network in a particular configuration 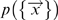 is then considered. The entropy *H* is then defined,
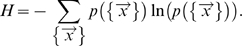
(S2)


Since it is apparent that the magnitude of fluctuations about the average of each time can greatly differ, data are rescaled to unit variance to focus on the relative shapes of the time courses. Therefore, a new set of scaled variables is considered:
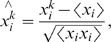
(S3)where brackets denote an average over *M* samples, and the k superscript denotes the k^th^ measurement and runs through each condition from 1 to 7 and denotes the k^th^ time point or measurement. When written in this form, 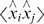 is the Pearson covariance matrix. Since our interest is in network connectivity, we consider two point (i.e. pairwise) interactions and therefore the mean and covariance of the data. The task is to maximize the entropy H subject to the constraints:
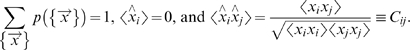
(S4)


For each element of the covariance matrix 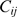, there is a corresponding Lagrange multiplier 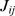 such that the procedure results in the following form for 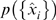:
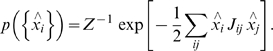
(S5)



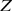
 is a normalization factor that can easily be obtained using standard methods but is not necessary for the purpose of this study. In future work, it may be interesting to study thermodynamic properties of the model by studying the behavior of 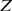 and its logarithm that constitutes a free energy. Also, higher order networks can be obtained by constraining the entropy to higher order moments of the probability distribution. Since the form of the resulting distribution 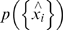 is a multivariate Gaussian whose argument (or energy function) is a summation over all pairwise and self interactions. The interaction matrix 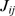 defined as 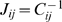, gives the pairwise coupling between each phosphorylation site in the network and is the subject of the analysis.

Since there are many more observed phosphorylation sites than independent samples of the network configuration, the problem of finding the unique network connectivity is ill-posed. Instead, the question that is asked is how to calculate the probability of a network configuration (i.e. phosphorylation state of the N Tyrosine sites that are measured). From the mass spec data[8], times courses of the relative amount of phosphorylation at each of 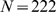 phosphorylated tyrosine sites is obtained at 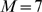 time points at times 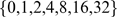 minutes. All data were normalized to unit variance and those phosphorylation sites having a small (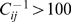) variance, on the order of the error bars in the experiments, were not considered. Since the resulting matrices are underdetermined (i.e. 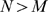), we obtain the interaction matrix by inverting 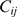 in the space of non-zero eigenvalues[16].

First, 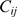 is expanded in terms of its eigenvalues and coefficients of its eigenvectors:
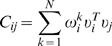
(S6)where the *T* denotes the transpose of the eigenvector 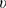. The matrix element 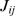 is obtained by inverting *C* in the space of eigenvectors containing non-zero eigenvalues by considering only the non-zero eigenvalues of 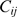:
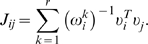
(S7)



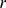
 is the rank of the covariance matrix. From the expansions in eqs. S6 and S7, it is seen that the correlations are dominated by the largest eigenvalues whereas the interactions are dominated by the smallest eigenvalues. The matrix elements 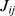 are interpreted as the residual, pairwise interactions between phosphotyrosine (pY) sites i and j that remain once global effects that are dominated by the correlations (i.e. the largest eigenvalues) are effectively removed. 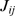 constitutes the matrix of pairwise network interactions. In this scenario, the interaction at sites i and j is not necessarily revealed by the correlation since other factors aside from pairwise interactions are influencing the correlation. Fig. S1 considers a plot of the histogram of values of 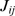 (Fig. S1a) and a plot of the full matrix 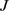 (Fig. S1b).

From the plot in Fig. S1a, it is apparent that the distribution of 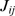 is sharply peaked about zero suggesting that most elements of the matrix contain no information. We therefore focused our analysis on the large magnitude interactions of 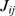. To show the difference in the measured covariances with the inferred interactions, Fig. S2 contains a scatter plot of the elements of 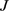 plotted against the elements of *C*. From the scatter plot, it is apparent that a complex relationship between the interactions and corresponding correlations exists.

Also note that since the data are mean-centered and scaled to unit variance, phosphorylation sites with small variance (across different data points) contribute more to the network structure. Therefore, sites with small variance (on the order of the error bars of the experiment) are not considered; for example, the Tyrosine of GSK3-β is constitutively phosphorylated and is not considered. The inverse of the variance (across different data points) at each site is also shown in Table 1 and in Table 2.

### Application of maximum entropy derived network to a model biochemical signaling network

The model of the signaling network consisting of a set of 105 coupled ODEs can be written as follows:
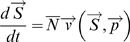
(S8)where 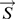 is an n-component concentration vector, 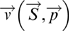 is a 110 component flux vector that is a function of 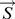 and parameter set 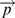, and 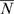 is a stoichiometry matrix whose columns consist of the chemical species and rows consist of chemical reactions corresponding to each of the fluxes. Eq. S8 can be solved using standard procedures. We chose to study this model because the transient behaviors in the calculated time courses roughly resemble those seen in the experimental data.

From the time-averaged solution of eq. S8, the elements of a time-averaged covariance matrix can be obtained:
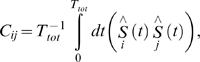
(S9)where 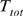 is the length of the time course, 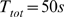, and the ^ denotes mean-centering and normalization to unit variance of the time course. Inverting 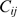 gives the interaction matrix 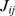 as contained in eq. 1. Introducing a threshold *T* as in eq. 3 defines the elements of a calculated connectivity matrix 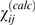,
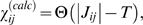
(S10)where 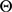 is a step function.

We then compared 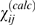 to a model network connectivity that can be computed from the stoichiometry matrix 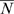. We defined an undirected network connectivity, 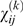 (of node distance k which we define below) from 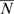 in the following way: we first considered a 1-point connectivity from the relation
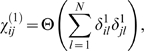
(S11)where 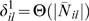 (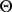 is a step function) and *N* is the number of reactions. That is, we sum over the rows of 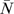 and look for two-point combinations of non-zero elements (two chemical species involved in the same reaction). Defined in this way, 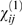 defines a network of pairs of chemical species that share a common chemical reaction. The k-point (k>1) network can then be obtained directly from the 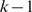 point network,
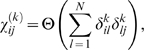
(S12)where 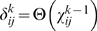, and 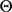 is a step function. Therefore, 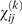 are elements of a Boolean matrix that are non-zero if species i and j are linked within any sequence of k half reactions. We note that 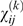 pertains only to the situation that considers the simulated system and does not bear any relation to the phosphorylation data.

Fig. 1a. compares the calculated 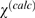
and 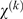 for k = 1,2,3,4. The fraction of correct interactions 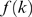 is computed by comparing all non zero elements of 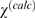
to their counterparts in 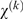; that is,
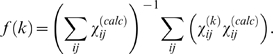
(S13)


For reference, the simulated model[20] contains 20 unique chemical species and therefore 190 possible interactions. Although 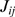 is undirected (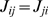), chemical reaction networks are defined by sequences of half reactions and therefore are directed networks. As a result, 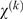 overcounts the total number of real interactions. For example in the half reaction 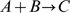, the species *A* and *B* are considered connected at a distance 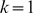 although their activities do not influence each other. We chose this convention because we compared two undirected networks. Thus, 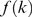 (Fig. 1a) overcounts the number of expected interactions by roughly a factor of 2 – in part, the reason for poor accuracy at node distance one is an artifact of the topology calculation.

### Affinity dependent network topology

Fig. 3 illustrates the dependence of the calculated network topology on choice of *T*. In Fig. S4, the node distribution 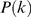 is plotted for four different values of *T*. 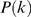 is defined as the probability that a pY site is interacting with *k* separate sites. At high thresholds (e.g. 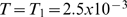) most signaling nodes have no connections. At low threshold (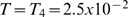) each phosphotyrosine site is connected to many other sites. At intermediate values of *T* (e.g. 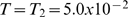 or 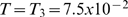), 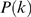 appears as a monotonically decaying, continuous function of *k*. Unfortunately, due to the insufficient number of samples of *k*, the large *k* behavior of 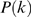 is not resolved. It is apparent, however, that at these intermediate threshold values, the presence of a significant tail of the distribution 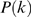 is apparent. At intermediate threshold values, 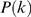 is seen as a monotonically decaying function *k*.

### Computation of network diagrams and topological properties

All networks shown in the text were drawn using the Cytoscape software package[29]. Topological properties such as the mean number of nearest neighbors and the average clustering coefficients were computed using the NetworkAnalyzer[30] plugin in the Cytoscape package.

Random networks were computed as follows: for a random network (Fig. 4a), the probability that a bond exists between two nodes for a choice of *T* is 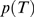. 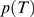 is taken to be 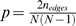 where 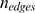 is the number of computed edges at threshold *T* and *N* is the number of nodes at *T*. In Fig. 1a, the fraction of correct interactions is taken to be 
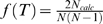
 where 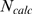 is the number of calculated bonds.

## Supporting Information

Figure S1 (0.12 MB DOC)

Figure S2 (0.51 MB DOC)

Figure S3 (0.08 MB DOC)

Figure S4 (0.54 MB DOC)

Figure S5 (0.07 MB DOC)

## References

[1] Pawson T (2004). Specificity in signal transduction: From phosphotyrosine-SH2 domain interactions to complex cellular systems.. Cell.

[2] del Sol A, Arauzo-Bravo MJ, Moya DA, Nussinov R (2007). Modular architecture of protein structures and allosteric communications: potential implications for signaling proteins and regulatory linkages.. Genome Biology.

[3] Ma BY, Nussinov R (2009). Amplification of signaling via cellular allosteric relay and protein disorder.. Proceedings of the National Academy of Sciences of the United States of America.

[4] Hunter T (2000). Signaling - 2000 and beyond.. Cell.

[5] Zhang Y, Wolf-Yadlin A, Ross PL, Pappin DJ, Rush J (2005). Time-resolved mass spectrometry of tyrosine phosphorylation sites in the epidermal growth factor receptor signaling network reveals dynamic modules.. Molecular & Cellular Proteomics.

[6] Olsen JV, Blagoev B, Gnad F, Macek B, Kumar C (2006). Global, in vivo, and site-specific phosphorylation dynamics in signaling networks.. Cell.

[7] Wolf-Yadlin A, Kumar N, Zhang Y, Hautaniemi S, Zaman M (2006). Effects of HER2 overexpression on cell signaling networks governing proliferation and migration.. Molecular Systems Biology.

[8] Wolf-Yadlin A, Hautaniemi S, Lauffenburger DA, White FM (2007). Multiple reaction monitoring for robust quantitative proteomic analysis of cellular signaling networks.. Proceedings of the National Academy of Sciences of the United States of America.

[9] Nielsen UB, Cardone MH, Sinskey AJ, MacBeath G, Sorger PK (2003). Profiling receptor tyrosine kinase activation by using Ab microarrays.. Proceedings of the National Academy of Sciences of the United States of America.

[10] Janes KA, Yaffe MB (2006). Data-driven modelling of signal-transduction networks.. Nature Reviews Molecular Cell Biology.

[11] Kumar N, Wolf-Yadlin A, White FM, Lauffenburger DA (2007). Modeling HER2 effects on cell behavior from mass Spectrometry phosphotyrosine data.. Plos Computational Biology.

[12] Janes KA, Gaudet S, Albeck JG, Nielsen UB, Lauffenburger DA (2006). The response of human epithelial cells to TNF involves an inducible autocrine cascade.. Cell.

[13] Sachs K, Perez O, Pe'er D, Lauffenburger DA, Nolan GP (2005). Causal protein-signaling networks derived from multiparameter single-cell data.. Science.

[14] Basso K, Margolin AA, Stolovitzky G, Klein U, Dalla-Favera R (2005). Reverse engineering of regulatory networks in human B cells.. Nature Genetics.

[15] Cover TM, Thomas JA (2006). Elements of Information Theory: Wiley.

[16] Lezon TR, Banavar JR, Cieplak M, Maritan A, Fedoroff NV (2006). Using the principle of entropy maximization to infer genetic interaction networks from gene expression patterns.. Proceedings of the National Academy of Sciences of the United States of America.

[17] Schneidman E, Berry MJ, Segev R, Bialek W (2006). Weak pairwise correlations imply strongly correlated network states in a neural population.. Nature.

[18] Zhan LX, Rosenberg A, Bergami KC, Yu M, Xuan ZY (2008). Deregulation of Scribble Promotes Mammary Tumorigenesis and Reveals a Role for Cell Polarity in Carcinoma.. Cell.

[19] Cantley LC (2002). The phosphoinositide 3-kinase pathway.. Science.

[20] Maeda A, Ozaki Y, Sivakumaran S, Akiyama T, Urakubo H (2006). Ca2+-independent phospholipase A2-dependent sustained Rho-kinase activation exhibits all-or-none response.. Genes to Cells.

[21] Le Novere N, Bornstein B, Broicher A, Courtot M, Donizelli M (2006). BioModels Database: a free, centralized database of curated, published, quantitative kinetic models of biochemical and cellular systems.. Nucleic Acids Research.

[22] Albert R, Barabasi AL (2002). Statistical mechanics of complex networks.. Reviews of Modern Physics.

[23] Audebert S, Navarro C, Nourry C, Chasserot-Golaz S, Lecine P (2004). Mammalian scribble forms a tight complex with the beta PIX exchange factor.. Current Biology.

[24] Shimomura Y, Aoki N, Ito K, Ito M (2003). Gene expression of Sh3d19, a novel adaptor protein with five Src homology 3 domains, in anagen mouse hair follicles.. Journal of Dermatological Science.

[25] Kittler R, Putz G, Pelletier L, Poser I, Heninger AK (2004). An endoribonuclease-prepared siRNA screen in human cells identifies genes essential for cell division.. Nature.

[26] Six DA, Dennis EA (2000). The expanding superfamily of phospholipase A(2) enzymes: classification and characterization.. Biochimica Et Biophysica Acta-Molecular and Cell Biology of Lipids.

[27] Pin JP, Galvez T, Prezeau L (2003). Evolution, structure, and activation mechanism of family 3/C G-protein-coupled receptors.. Pharmacology & Therapeutics.

[28] Alon U (2007). An Introduction to Systems Biology: Design Principles of Biological Circuits: Chapman & Hall.

[29] Shannon P, Markiel A, Ozier O, Baliga NS, Wang JT (2003). Cytoscape: A software environment for integrated models of biomolecular interaction networks.. Genome Research.

[30] Assenov Y, Ramirez F, Schelhorn SE, Lengauer T, Albrecht M (2008). Computing topological parameters of biological networks.. Bioinformatics.

