# Supplementary material for: Maximum Entropy Reconstructions of Dynamic Signaling Networks from Quantitative Proteomics Data
Source: PLoS One. 2009 Aug 26;4(8):e6522. doi: 10.1371/journal.pone.0006522 (PMC2728537; doi:10.1371/journal.pone.0006522)
Supplement: Figure S1 — (0.12 MB DOC) [file pone.0006522.s001.doc]

Figure S1. **Pairwise network interactions**

a.) $P\left(J_{ij}\right)$is plotted as a function of $J_{ij}$. $P\left(J_{ij}\right)$is sharply peeked at zero. b.) Contour plot of the interaction matrix $J$.
