# Supplementary material for: Maximum Entropy Reconstructions of Dynamic Signaling Networks from Quantitative Proteomics Data
Source: PLoS One. 2009 Aug 26;4(8):e6522. doi: 10.1371/journal.pone.0006522 (PMC2728537; doi:10.1371/journal.pone.0006522)
Supplement: Figure S2 — (0.51 MB DOC) [file pone.0006522.s002.doc]

Figure S2. **Scatter plot of calculated $J$vs** $C$

Each matrix element $J_{ij}$is plotted against $C_{ij}$. A complicated relation is observed that suggests that $C_{ij}$and $J_{ij}$ have different meanings
