# Supplementary material for: Maximum Entropy Reconstructions of Dynamic Signaling Networks from Quantitative Proteomics Data
Source: PLoS One. 2009 Aug 26;4(8):e6522. doi: 10.1371/journal.pone.0006522 (PMC2728537; doi:10.1371/journal.pone.0006522)
Supplement: Figure S3 — (0.08 MB DOC) [file pone.0006522.s003.doc]

Figure S3. **Self interactions and network hubs**

a.) values of self interactions. Value of $J_{ii}$ for each of the N=222 is plotted against phosphorylation (pY) site number. b.) values of inverse variance $\left(C_{ii}\right)^{-1}$are shown for each pY site. Large values $J_{ii}$ indicate large contributions to the overall network. Nodes with large$\left(C_{ii}\right)^{-1}$($\left(C_{ii}\right)^{-1}>100.0$) were ignored since their variations are small and too close to the error bars in the experiment.
