# Supplementary material for: Maximum Entropy Reconstructions of Dynamic Signaling Networks from Quantitative Proteomics Data
Source: PLoS One. 2009 Aug 26;4(8):e6522. doi: 10.1371/journal.pone.0006522 (PMC2728537; doi:10.1371/journal.pone.0006522)
Supplement: Figure S4 — (0.54 MB DOC) [file pone.0006522.s004.doc]

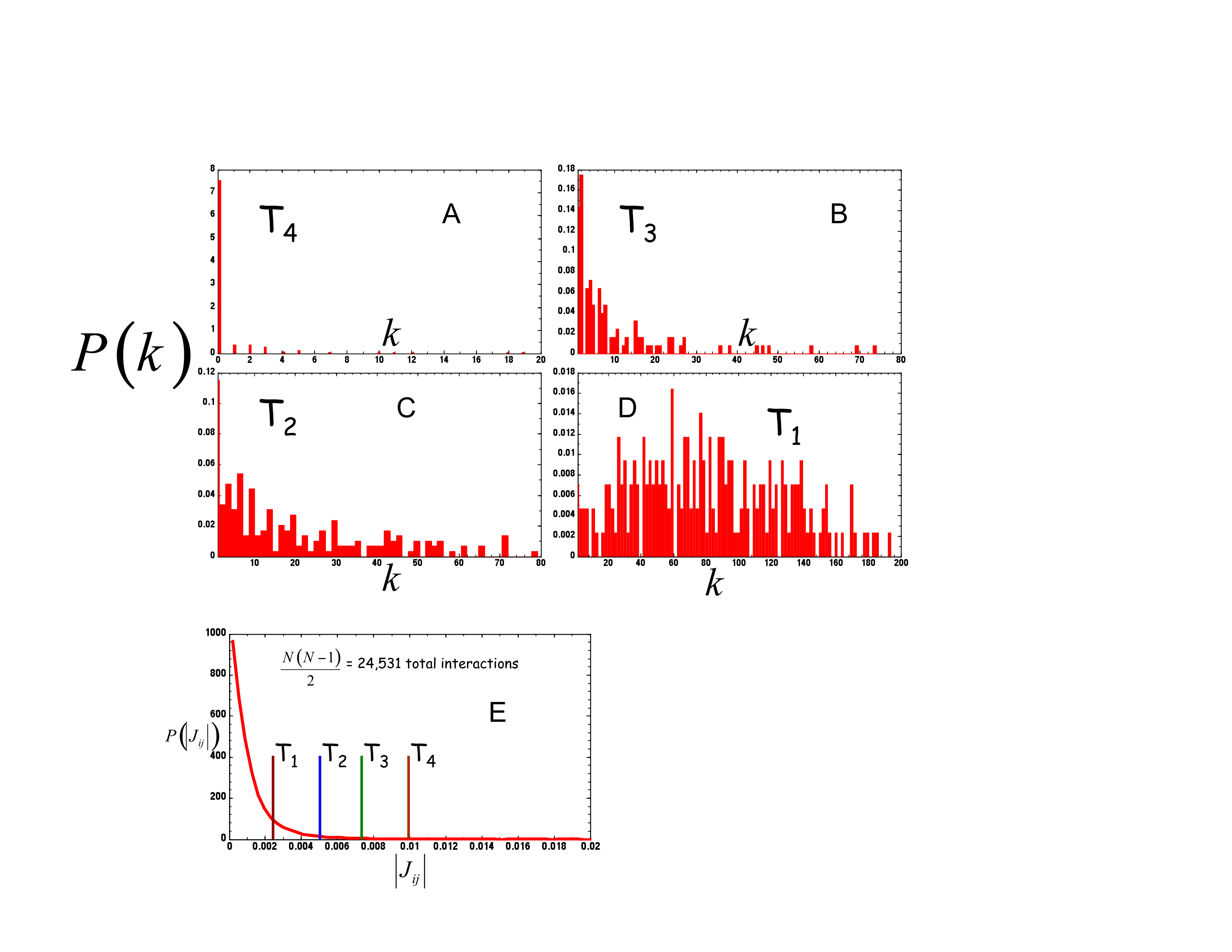


Figure S4. **Threshold dependence of node distribution**

The node distribution (i.e. probability that a phosphorylation site is linked to k other sites) $P\left(k\right)$is plotted for four values of the threshold $T_{k}$; an interaction between sites i and j is counted if a.) $T_{4}=10.0x10^{-2}$, b.) $T_{3}=7.5x10^{-2}$, c.) $T_{2}=5.0x10^{-2}$, d.) $T_{1}=2.5x10^{-3}$. e.) histogram of with the locations of different thresholds denoted.
