# Supplementary material for: Maximum Entropy Reconstructions of Dynamic Signaling Networks from Quantitative Proteomics Data
Source: PLoS One. 2009 Aug 26;4(8):e6522. doi: 10.1371/journal.pone.0006522 (PMC2728537; doi:10.1371/journal.pone.0006522)
Supplement: Figure S5 — (0.07 MB DOC) [file pone.0006522.s005.doc]

Figure S5. **Graphical depictions of the network connectivity of the three highest scoring uncharacterized phosphorylation sites.**

Network connectivity of phosphorylation sites associated with proteins a.) ARHGEF7 b.) SH3D19, and c.) ZDHHC5 A threshold value T = 0.05 is used to define interactions centered on each node for each phosphorylation site.
